# News and Events of the Week

**Published:** 1911-02-25

**Authors:** 


### News and Events of the Week.

Two lectures will be given at the Central London Throat and Ear Hospital, Gray's Inn Road, W.C., on Tuesday, February 28, and Friday, March 3, at 3.45 p.m., by Mr. Chichele Nourse, on "Accessory Sinuses."

The budget of the "Assistance Publique," which in France to some extent takes the place of our system of poor relief, has been estimated for this year at 81,134,184 francs (£3,245,367), of which sum the city of Paris will be called upon to provide 25,216,950 francs (£1,008,678).

The Third International Congress for the Protection of Childhood from Infancy, "Goutte de Lait," will be held at Berlin from September 11 to September 15, under the presidency of Professor Dietrich, of Berlin. The general secretary is Professor Keller, of Charlottenburg.

The following are the arrangements for next week at the West London Postgraduate College, West London Hospital, Hammersmith Road : February 27 and March 2, at 10 a.m., Demonstration by Surgical Registrar; February 27, at 12 noon, Pathological Demonstration; February 28, at 11.30 a.m., Demonstration of Minor Operations; March 1, at 10 a.m., Gynæcological Demonstration; and March 2, at 12.15 p.m., Lecture, Dr. Grainger Stewart, "Practical Medicine." Lectures at 5 p.m. : February 27, Dr. Morton, "Therapeutics"; February 28, Dr. Seymour Taylor, "Some Medical Aphorisms"; March 1, Dr. Beddard, "Practical Medicine" (Lecture V.); March 2, Dr. Drummond Robinson, "Gynæcological or Obstetrical Subject"; and March 3, Dr. Abraham, Cases of Skin Disease.

Dr. Henri Huchard, whose obituary notice recently appeared in these columns, has bequeathed a sum of 100,000 francs (£4,000) to the Paris Academy of Médecine to found an annual prize to be awarded by that body. The prize is to be named "The Prize of Henri Huchard of the Academy of Médecine.—A prize for medical devotion in remembrance of Marcel Huchard." The donor intended by founding this prize to come to the help of young students who, as in the case of his own son, were the victims of professional devotion, so as to enable them to continue their studies.

The following is a list of the articles contained in the new emergency medical outfit approved by the French military authorities : (*a*) A metal box containing a michel holder and fifty clips; (*b*) a metal box made to contain tubes of tablets of antipyrine, quinine, ampoules of morphine and caffeine, a tube of ethyl chloride, etc. This list of drugs can be increased as the surgeon wishes. (*c*) A metal box for surgical instruments as follows : two folding bistouris, two hæmostatic forceps (Doyen's) capable of serving as needle-holders, one pair of straight scissors with rounded ends, one skein of silk contained in a glass tube protected by a metal case, one metal box containing twenty assorted needles for suturing, one hypodermic syringe, one hollow sound, one silver male and female catheter joined by a screw, one pair of dissecting forceps, one clinical thermometer; (*d*) one hæmostatic band (Denain's); (*e*) two sets of dressings. There are all contained in a morocco wallet, closed by a metal clasp. Every military surgeon is obliged to carry this outfit with him on all occasions, whether in hospital or in the field, on which he is in charge of troops.

The annual meeting of the After-Care Association for poor persons discharged recovered from Asylums for the Insane was held on Wednesday, February 8, by the kind permission of Sir Victor and Lady Horsley, at 25 Cavendish Square, Mr. H. D. Greene, K.C., presiding over a large audience. The report read by Mr. Roxby stated that applications were received during 1910 on behalf of 379 cases, and the total income amounted to £1,199 12s. 6d. The adoption of the report was moved by the Bishop of Barking, seconded by Dr. G. H. Savage, supported by the Master of the Temple and Dr. Robert Jones (Claybury Asylum). The re-election of Vice-Presidents, Council, etc., was proposed by the Rev. H. R. Gamble, Vicar of Holy Trinity, Chelsea, seconded by Dr. Bond (Long Grove Asylum) and supported by Dr. Stoddart (Bethlehem Royal Hospital). A vote of thanks to the Chairman terminated a most successful meeting.

The International Committee for Post-Graduate Medical Education, which held a meeting in October 1910, is at present occupied in the execution of two tasks decided upon at the meeting, which are of interest to the medical men of all countries. The first problem relates to the organisation of an establishment for the collection of material to enable the already existing information bureau of the Kaiserin Friedrich-Haus in Berlin to furnish information at all times gratis to physicians, regarding the opportunity for post-graduate medical education in the different civilised countries. The establishment is to be a permanent one, and therefore the necessary researches are to be undertaken at regular intervals of a half-year. The second problem is the arrangement of a collective investigation in the departments of medical instruction at the universities and post-graduate medical education, and has reference to the following subjects : A. Academic University Instruction. B. Post-Graduate Medical Education. I. Organisation of the Post-Graduate Medical Education (including statements in regard to time, place, and the question of fees). (1) Associations for Post-Graduate Medical Education (Statutes)); (2) Post-Graduate Medical Education at the University; (3) Post-Graduate Medical Education and University instruction in their reciprocal relations and limitations. II. Special Institute for Post-Graduate Medical Education : (1) Clinical or Polyclinical Institutes; (2) Academies (and similar teaching institutes). C. Medical Educational Appliances.

The following are the arrangements for next week at the Medical Graduates' College and Polyclinic, 22 Chenies Street, W.C. : Monday, February 27, at 4 p.m., Dr. J. H. Sequeira, Clinique (skin); at 5.15 p.m., Dr. Eric Pritchard, "The Treatment of Common Symptoms among Infants." Tuesday, at 4 p.m., Dr. Guthrie Rankin, Clinique (Med.); at 5.15 p.m., Dr. James Taylor, "Eye Affections in Nervous Diseases." Wednesday, March 1, at 4 p.m., Sir A. Pearce Gould, Clinique (Surg.); at 5.15 p.m., Dr. Frederic Taylor, "Anæmia." Thursday, at 4 p.m., Mr. James Berry, Clinique (Surg.); at 5.15 p.m., Dr. E. C. Hort, "The Use and Abuse of Tuberculin." Friday, at 4 p.m., Mr. W. Stuart-Low, Clinique (Ear, Nose and Throat).

A Fifth edition of Mr. Bland-Sutton's "Tumours : Innocent and Malignant," incorporating much new matter, is announced by Messrs. Cassell and Company for publication on March 10.

A mural tablet has been erected in the cloisters of Santa Croce at Florence to the memory of the late Miss Nightingale. Miss Nightingale was born at Florence in Villa Colombaia, where a similar memorial has been placed.

The International Congress of Pædology will be held at Brussels during the holidays of 1911. Dr. Desguin, member of the Royal Academy of Médecine, has accepted the presidency of the Belgian Committee. All information can be obtained from the Congress Committee, 141 rue Brèderode, Brussels.

The Council of the London and Counties Medical Protection Society, at its meeting on January 18 passed the following resolution:—"The Council of this Society, on the unanimous recommendation of its Dental Committee, after duly considering 'an Act to amend the Dentists Act, 1878, and for other purposes in connection therewith,' promoted by certain members of the British Dental Association, strongly disapproves of it;' and urges its Dental members to offer strenuous opposition to this or any other Act by which unqualified practice may be legalised."

